# Cellular models of pain: New technologies and their potential to progress preclinical research

**DOI:** 10.1016/j.ynpai.2021.100063

**Published:** 2021-05-21

**Authors:** Lina Chrysostomidou, Andrew H. Cooper, Greg A. Weir

**Affiliations:** Institute of Neuroscience and Psychology, College of Medical, Veterinary and Life Sciences, University of Glasgow, Glasgow, UK

**Keywords:** Sensory neuron, Pain, Nociception, Human dorsal root ganglion, Stem cell-derived sensory neuron

## Abstract

In vitro models fill a vital niche in preclinical pain research, allowing detailed study of molecular pathways, and in the case of humanised systems, providing a translational bridge between in vivo animal models and human patients. Significant advances in cellular technology available to basic pain researchers have occurred in the last decade, including developing protocols to differentiate sensory neuron-like cells from stem cells and greater access to human dorsal root ganglion tissue. In this review, we discuss the use of both models in preclinical pain research: What can a human sensory neuron in a dish tell us that rodent in vivo models cannot? How similar are these models to their endogenous counterparts, and how should we judge them? What limitations do we need to consider? How can we leverage cell models to improve translational success? In vitro human sensory neuron models equip pain researchers with a valuable tool to investigate human nociception. With continual development, consideration for their advantages and limitations, and effective integration with other experimental strategies, they could become a driving force for the pain field's advancement.

## Highlights

• Human sensory neurons can reduce the translational gap in analgesic development.
• Access to dorsal root ganglion (hDRG) neurons is increasing.
• Diverse sensory neuron subtypes can now be generated via stem cell technology.
• Advances of these technologies will improve our understanding of human nociception.

## Introduction

Peripheral sensory neurons are the initial transducers of noxious stimuli; however, they are only the first relay in the complex pain pathway, which involves diverse cell types, intricate spinal cord circuitry, a fine balance of ascending and descending neural traffic, and coordinated recruitment of many brain regions. This point is made clear in the recent update of the International Association for the Study of Pain’s definition of pain, which states that, “Pain cannot be inferred solely from activity in sensory neurons” (Raja et al., 2020). It is right that *in vivo* models that encapsulate all of the aforementioned complexity take a prominent role in preclinical pain research. What then is the utility of studying *in vitro* sensory neuron models of pain?

**Fig. 1:**
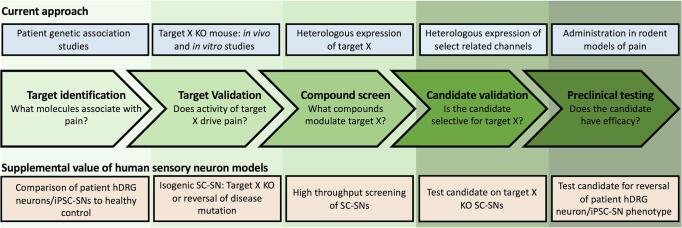
Analgesic drug discovery pathway and the utility of hDRG neurons and stem cell-derived sensory neurons (SC-SNs). Drug development includes several stages prior to clinical development. The illustration depicts key stages of development from the perspective of a candidate drug target, target X, found to associate with chronic pain. Top row provides example experimental approaches that are currently used at each stage. Bottom row details the potential for SC-SNs and hDRG neurons to supplement preclinical drug development and increase success rates. KO- knockout.

There are two fundamental reasons why studying cellular systems is a productive use of preclinical pain research resources. Firstly, there are practical advantages of reducing the experimental unit from animal to cell: reduced expense, ethical concern and experimental labour, and ease of manipulation. These points make a case for using cell models in experimental scenarios where large numbers of conditions or treatments are tested, such as drug screening. Secondly, differences in species physiology are often postulated to explain why novel analgesic compounds can generate promising preclinical data but fail to demonstrate clinical efficacy. Recent comparison of mouse and human dorsal root ganglion (DRG) tissue lends credence to this logic (Rostock et al., 2018, Shiers et al., 2020). Human cell models offer a supplemental approach to validate drug targets along the translational pathway before precious research capital is expended on clinical development (Fig. 1).

There has been a rapid acceleration in the development of high-quality human sensory neuron models in the last decade, including stem cell technology and DRG neurons from human donors (hDRG neurons). Improvements in model representation and increased availability make the use of *in vitro* models more attractive. There is a concomitant need to evaluate their appropriate place in preclinical pain research, develop agreed standards of practise, and acknowledge limitations. Here, we review recent advances in human sensory neuron models, examine their current use in investigating pain biology, and highlight several opportunities for future use.

## Endogenous hDRG neurons

**Fig. 2:**
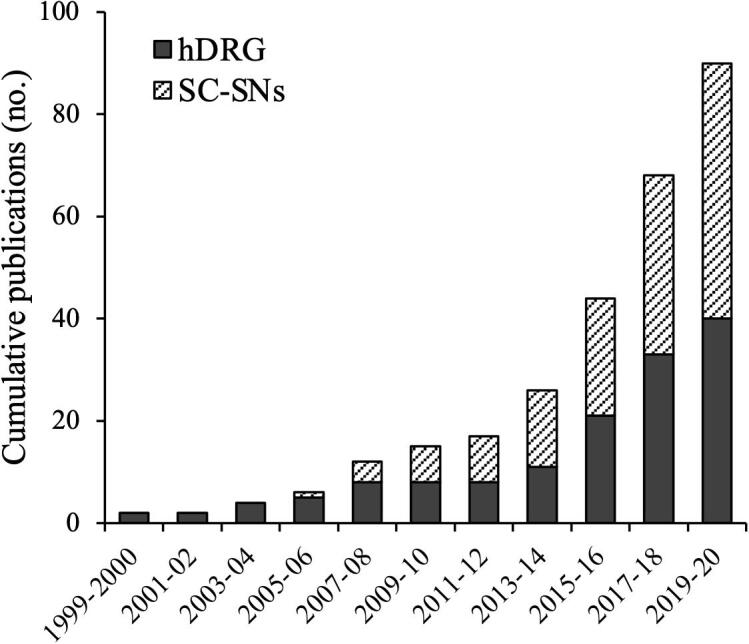
Expanding use of hDRG neurons and SC-SNs. The chart highlights the increase in hDRG neurons and SC-SNs use in the last two decades. Publications were discovered in PubMed by the search terms, “cultured human DRG neurons”, “hDRG neuron culture” and “stem cell” “sensory neurons”, “iPSC sensory neurons” and “embryonic stem cell sensory neurons” for hDRG neurons and SC-SNs respectively. Publications relating to topics other than somatosensation or those differentiating multipotent cells were not included.

hDRG neuron cultures have been used in translational pain studies for nearly 25 years. Their use, however, has been constrained by availability and predominantly relied on neurons derived from fetal tissue. Greater access to high-quality hDRG from previously healthy post-mortem organ donors, and patients undergoing spinal surgery for disease treatment, has led to a substantial increase in use over the last five years (​Fig. 2). This in turn has spurred the development and optimisation of culturing methods (​Valtcheva et al., 2016​), enabling and encouraging broader use of hDRG neurons in preclinical research. However, given the persisting difficulty of obtaining hDRG neurons, do they represent a worthwhile investment of resources over rodent sensory neuron cultures?

## hDRG neurons; an advance on rodent sensory neurons?

Sensory neurons dissociated from mouse or rat DRG have, and continue to be, the predominant *in vitro* model in preclinical pain research. Findings from such studies have been pivotal for our understanding of nociceptive signalling pathways and identifying potential drug targets. However, many fields of medical research are increasing their use of human-based systems to avoid species differences and mitigate failures in translation. Transcriptional studies of whole DRG tissue demonstrate similarities, but also a substantial divergence of gene expression between rodent and human DRG (Chang et al., 2018, Ray et al., 2018, Rostock et al., 2018, Shiers et al., 2020). For instance, neurofilament 200 (NF200) is a canonical marker of medium/large myelinated neurons in rodents, whereas virtually all DRG neurons express it in humans (Chang et al., 2018, Rostock et al., 2018). Similarly, CGRP, a population-specific marker of peptidergic afferents in mouse, is more widely distributed in human (Shiers et al., 2020).

Therefore, a primary use of cultured hDRG neurons is to test whether the wealth of data amassed on basic rodent sensory neuron physiology also applies to humans. Encouragingly, many neuronal properties are evolutionarily conserved. The action potential waveform of small and medium-sized hDRG neurons shares characteristics with rodent, such as an inflection on the falling phase and extended after-hyperpolarisation (AHP) (Davidson et al., 2014). hDRG neurons are responsive to classical algogens, pruritogens, and inflammatory mediators (Anand et al., 2008, Davidson et al., 2014, Zhang et al., 2017) and engage some common fundamental analgesic signalling pathways (Davidson et al., 2016, Moy et al., 2020). Despite the similarities, discrepancies in the response profile of several potential analgesic targets exist. Differences in the pharmacology and activated current kinetics have been observed for GABA_A_ (Zhang et al., 2015) and nicotinic acetylcholine receptors (Zhang et al., 2019), and voltage-gated Ca^2+^ channels (Moy et al., 2020). Additionally, whilst activation of metabotropic glutamate receptors 2/3 (mGluR2/3) blocks prostaglandin E_2_ (PGE_2_)-induced nociceptor membrane hyperexcitability in both human and mouse (Davidson et al., 2016), it attenuates PGE_2_-induced TRPV1 sensitisation in mouse but not human (Sheahan et al., 2018).

Voltage-gated Na^+^ channels (VGSCs) play an important role in sensory neuron excitability and constitute a significant focus of preclinical pain research (Bennett et al., 2019). A recent comparison of the biophysical and pharmacological properties of VGSCs in rat and human sensory neurons neatly demonstrated the feasibility of garnering substantial data sets from hDRG neurons (Zhang et al., 2017). The authors of this study concluded that while the tetrodotoxin (TTX)-sensitive (TTX-S) and TTX-resistant (TTX-R) currents are qualitatively similar between the two species, several differences exist, which could impact drug development (Zhang et al., 2017). The ratio of TTX-R to TTX-S was reduced in human DRG neurons, however, TTX-R was present in even the largest diameter neurons. This is in contrast to rodent DRG neurons, in which the presence of TTX-R is enriched in small diameter neurons (Djouhri et al., 2003). VGSC pharmacology was markedly different to that observed when studied in rodent sensory neurons and heterologous expression of human VGSCs. These findings, detailing divergence in proposed analgesic targets' properties, reinforce the value of using hDRG as a validation step in the translational pathway.

A second utility of hDRG neurons is to test whether analgesic strategies validated in rodent models can work in human tissue. This is highly attractive because it acts as a time and cost-effective means of giving further confidence in future clinical testing, potentially reducing the wastage associated with failed clinical trials. Toll-like receptor 5 (TLR5) is expressed in large diameter A-fibres. Receptor activation permits entry of the membrane impermeable QX-314 (a lidocaine derivative), resulting in A-fibre conduction block and reversal of mechanical hypersensitivity in rodent models of neuropathic pain (Xu et al., 2015). The approach showed comparable effectiveness in hDRG neurons. Selective inhibitors of Na_V_1.7 developed using mouse DRG and heterologous systems and proposed as analgesic compounds, also function similarly in hDRG neurons (Alexandrou et al., 2016). These works support translatability by demonstrating that the target receptor/channel is present in the appropriate hDRG neurons and can be similarly manipulated. Proof of principle studies extend to non-pharmacological approaches. Optogenetic neuromodulation has been proposed as a treatment modality for bladder dysfunction and interstitial cystitis (Mickle et al., 2019). While testing of the whole system requires *in vivo* models, the demonstration that activation of archaerhodopsin reduces neuronal excitability in rat and hDRG neurons in a similar manner is an important advance (Mickle et al., 2019). Besides validating analgesic targets, hDRG neurons likely offer an improved platform to assess pharmacological specificity and “off-target” effects. Just as the profile of “on-targets” differ between human, rodent and heterologous expression systems, so too may “off target” interactions (Sison-Young et al., 2017).

## hDRG neurons as a tool to understand chronic pain

Beyond validation, recent work has illustrated the promise hDRG neurons hold for understanding underlying pathomechanisms of chronic pain. For example, the application of neurotoxic agents onto healthy neurons can model neuropathic pain. Administration of the chemotherapeutic agent paclitaxel increases Na_V_1.7 expression, enhances neuronal excitability, and sensitizes TRPV1 via TLR4 activation (Chang et al., 2018, Li et al., 2015). Analogous experiments with different nerve insults (e.g. traumatic injury, hyperglycaemia, etc.) are undoubtedly possible and could yield important insights. The availability of hDRG neurons from patients with chronic pain represents an exciting avenue to directly explore environmental disease factors. Spontaneous activity (SA) of sensory neurons is a critical factor in initiating and maintaining neuropathic pain, as highlighted by rodent studies and microneurography recordings in patients (Serra et al., 2012, Wall and Gutnick, 1974). North *et al.* demonstrated that hDRG neurons from pain patients with a history of radicular/neuropathic pain also exhibit SA (North et al., 2019). Crucially, ectopic activity was only present in hDRG neurons harvested from painful dermatomes. Ectopically active hDRG neurons exhibited neurophysiological characteristics consistent with those seen in rodent models of neuropathic pain, including, a hyperpolarizing shift in the voltage threshold for action potential generation and membrane potential oscillations (Odem et al., 2018). Now that it is clear that disease relevant phenotypes can be retained following dissociation and culture, efforts can be directed to discovering underlying signalling pathways that may serve as novel therapeutic avenues. In the future, multi-institutional procurement networks (for example, as proposed by Renthal et al.(2021)) may be leveraged to coordinate tissue acquisition and permit correlations of pain phenotypes with physiological data at scale.

## Challenges and limitations

The use of hDRG neurons has already given important insights, however, several challenges surrounding their use need to be addressed before their potential is maximised. Despite increased availability, access to viable hDRG neurons remains limited to a few labs and the frequency of suitable donor availability varies depending on the institution and inclusion criteria. Consistency of donor characteristics (age, sex, genetic background, medical history etc.) and time from cessation of circulation to culture is straightforward in rodent studies, however, because of sample scarcity, the same cannot be said for human tissue (Moy et al., 2020). To what extent such factors impact hDRG neuron physiology remains an open question. Donor heterogeneity brings challenges but may also offer biological advantage. The limited genetic variability of inbred mouse lines is postulated to contribute to failures of replication (Tuttle et al., 2018). Findings derived from hDRG neurons of donors with diverse genetic backgrounds will likely be more generalisable across the population and may therefore result in improved translation. Furthermore, heterogenous environmental factors, such as medication history, can be leveraged to gain biological insight (Moy et al., 2020). Sex differences are being increasingly investigated in the field of pain, with evidence highlighting sexual dimorphisms in pain mechanisms and plasticity (Sorge et al., 2015, Vacca et al., 2014). hDRG neurons in culture may offer valuable insight on the topic. Global gene expression and basic electrophysiological parameters are similar between hDRG neurons derived from male and female donors (North et al., 2019, Wangzhou et al., 2020). However, differences have been observed in analgesic responses, as showcased by the increased responsiveness of male hDRG neurons to DAMGO, a selective µ-opioid agonist, compared to female neurons, which are more responsive to the δ-opioid agonist SNC80 (Moy et al., 2020). Increasing evidence supports sex-specific differences in sensory neuron interactions with the immune and endocrine systems (Lopes et al., 2017b, Paige et al., 2020). Therefore, co-culture systems containing hDRG and interacting cellular partners (discussed below) will likely be a valuable resource to dissect sexually dimorphic pain pathways.

Culture conditions, particularly the presence of additives in the media, have substantial influence on experimental findings. Further optimisation and standardisation of culturing conditions will help reduce inter-lab variability. Such inconsistencies could explain discrepancy in current data from different labs. For example, Zhang *et al.* observed reduced persistent TTX-R current in hDRG neurons and a reduced contribution of Na_V_1.7 to TTX-S current than in rodent DRG neurons, in contrast with findings from other groups with hDRG neurons (Alexandrou et al., 2016, Han et al., 2015). This variation may be attributed to differences in culture conditions. For instance, the latter two studies supplemented culture media with glial cell line-derived neurotrophic factor (GDNF), whilst Zhang *et al.* did not, and VGSC expression and function in cultured rodent sensory neurons is highly dependent on the cocktail of supplemented growth factors (Fjell et al., 1999, Leffler et al., 2002). Publishing detailed protocols and full datasets on open repositories will facilitate progress towards optimising culture conditions that more faithfully recapitulate native hDRG neurons, and will aid in future systematic analysis identifying factors (media, growth factors, time in culture, etc.) that have consequence for the biology under study.

Mechanical dissociation is a prerequisite of culture. Owing to their larger volume to surface area ratio, and hence reduced membrane durability, the process is known to bias for apoptosis of larger diameter neurons (Aboualizadeh et al., 2015, Lopes et al., 2017a). Some studies of hDRG cultures report neurons with smaller diameter relative to neurons measured *in situ*^8,13^, suggestive of loss of larger diameter neurons during dissociation. In future it will be important to assess the relative survival of each population type to confirm whether hDRG neuron cultures contain the full panoply of sensory neuron types. A further confound of dissociation is that the concomitant axotomy results in an injury-like, inflammatory phenotype (Valtcheva et al., 2016, Wangzhou et al., 2020). Dissociation-induced transcriptional changes include alterations in the abundance of GPCRs, receptor kinases and ion channels, and overlap with the transcriptional signature of neuropathic pain, and changes extend to some analgesic drug targets (Wangzhou et al., 2020). Additionally, recent transcriptomic studies have shown that *in vivo,* axotomy induces loss of sensory neuron identity, with neurons reverting to a common injured phenotype (Nguyen et al., 2019, Renthal et al., 2020). If this phenomenon occurs during dissociation and culture, study of discrete populations of hDRG neurons will be difficult. Cell stress during dissociation and culture will remain unavoidable, however, better knowledge of these changes will allow findings to be contextualized appropriately. Once technical challenges are overcome, comparisons of single-cell transcriptomic profiles of native and cultured hDRG neurons will provide a valuable insight, validating whether populations in culture correlate with the native state.

## Stem cell-derived sensory neurons (SC-SNs)

Sensory neuron-like cells have been generated from human embryonic stem cells (hESCs) (Chambers et al., 2012, Lee et al., 2007, Schrenk-Siemens et al., 2015) and induced pluripotent stem cells (iPSCs) (Chambers et al., 2012, Nickolls et al., 2020, Schrenk-Siemens et al., 2015). The advent of iPSC-technology, in particular, affords significant opportunities for pain modelling and analgesic screening. Following on rapidly from the initial description of the technology in 2007 (Takahashi et al., 2007), the pain field has been at the forefront of developing, validating, and utilising strategies to differentiate pluripotent cells into human sensory neurons. In comparison to hDRG neurons, they have two major advantages: the ability to study neurons with rare genetic defects, and the opportunity to derive an almost limitless supply of neurons for study. Donor cells are typically skin fibroblasts (Takahashi and Yamanaka, 2006) and the non-invasive manner of skin biopsies makes harvesting cells from patients with rare genetic disorders more realistic than obtaining donor hDRG for culture. Alternatively, iPSC are highly amenable to genome-engineering; rare mutations can be introduced to well-characterised healthy control lines, gene knockouts generated, or pathogenic mutations corrected to create isogenic control lines (Mazzara et al., 2020, McDermott et al., 2019, Nickolls et al., 2020). Pluripotent cells can be maintained and expanded in culture, allowing for vast numbers of cells for study. From one 3 mm skin punch biopsy, trillions of pluripotent cells can be generated, although vigilance is required to detect chromosomal instability induced by multiple rounds of passaging (Laurent et al., 2011). hDRG neurons rely on streamlined local organ procurement networks, whereas pluripotent stem cells can be cryopreserved and shipped to any laboratory with the expertise to culture.

## What sensory neuron subtypes can be generated?

Productive use of stem cell technology relies on differentiation protocols that accurately replicate somatic cell types. Dorsal root and trigeminal ganglia contain tens of molecularly distinct sub-populations of sensory neurons (Nguyen et al., 2017, Sharma et al., 2020, Usoskin et al., 2015), each contributing to different aspects of somatosensation (Arcourt et al., 2017, Cavanaugh et al., 2009, Dhandapani et al., 2018, Huang et al., 2018). While we cannot yet recreate the full panoply of sub-population diversity, there has been recent progress in expanding the list of populations that can be differentiated (Table 1).

**Table 1:** Sensory neuron differentiation strategies and resultant characteristics. Primary afferents can be classified into discrete functional subtypes (*shaded boxes*)**.** Stem cell differentiation (and transdifferentiation) is capable of producing cells with expression profile and/or functional features that overlap with most subtypes. It is important to note that perfect replication of endogenous neurons is unlikely. Protocols often derive heterogeneous cultures of SC-SNs, exhibiting features of multiple populations (highlighted by asterisk). Single-cell RNA sequencing (scRNAseq) data from mouse DRG (*bottom three rows*) illustrates that many endogenous subtypes contain several discrete sub-populations. While recent transcriptional profiling of non-human primate DRG neurons defined many of the described mouse subtypes (Kupari et al., 2021), it is unknown whether all sub-populations defined in mouse, are represented in human. More work is thus required to understand the exact sub-population which each protocol best represents and the degree to which each mirror endogenous counterparts. Protocols generating SC-SNs without functional testing of subtype profile are not listed. LTMR, low threshold mechanoreceptor; AP, action potential; RA, rapidly adapting; SA, slowly adapting; His, histamine; CQ, chloroquine. (See below-mentioned references for further information.)

The most commonly used protocol to comprehensively generate sensory neurons from iPSC was developed in 2012 (Chambers et al., 2012). A cocktail of small molecule inhibitors differentiates hiPSCs into a homogenous culture of neurons with a transcriptional and functional profile consistent with DRG nociceptors. These neurons exhibit a shoulder on the falling phase of the action potential waveform and TTX-R VGSC function, two hallmarks of endogenous rodent nociceptors (Blair and Bean, 2002, Ritter and Mendell, 1992). Differentiating neurons initially up-regulate and then down-regulate TRKA, and later exhibit robust RET expression. This developmental time-course is consistent with rodent non-peptidergic nociceptors (Luo et al., 2007). Supporting such a characterisation, neurons are highly responsive to the P_2_X_3_R agonist, α,β me-ATP. A large proportion of putative human nociceptors express TRPV1 (Shiers et al., 2020) and respond to capsaicin in culture (Baumann et al., 1996). While many neurons derived from the Chambers’ protocol express TRPV1 protein (Eberhardt et al., 2015), very few respond functionally to capsaicin (Chambers et al., 2012). An inability to engage such a major nociceptive signalling pathway is a drawback of this protocol and must be considered when interpreting results. Despite evidence for a C-fibre identity, neurons are readily myelinated when co-cultured with Schwann cells (Clark et al., 2017), making it difficult to rule out an Aδ-nociceptive identity. Several groups have adopted the Chambers protocol and report congruous findings (Eberhardt et al., 2015, McDermott et al., 2019, Mis et al., 2019, Pettingill et al., 2019, Schwartzentruber et al., 2018), however, there are also reports of the same protocol generating neurons with gene expression profiles resembling other sensory neuron types, such as pruriceptors (Umehara et al., 2020), C-low threshold mechanoreceptors (Guimarães et al., 2018) and proprioceptors (Dionisi et al., 2020). Such differences in resultant cell type likely result from minor protocol modifications and illustrate the differentiation sensitivity, especially when small molecules and defined media are employed. A more recent chemically-defined differentiation strategy generates neurons with expression profiles of nociceptors, mechanoreceptors and proprioceptors, and neatly demonstrates the potential for subtype enrichment by immunopanning (Saito-Diaz et al., 2021).

An alternative to small molecule differentiation, two publications in 2015 demonstrated that forced expression of canonical sensory neuron transcription factors can rapidly convert skin fibroblasts to functional sensory neurons (iSNs) (Blanchard et al., 2015, Wainger et al., 2015). The combination of factors employed biased for the differentiation of pure populations of nociceptors (Wainger et al., 2015), or a heterogenous group of neurons selective in their expression of TRKA, TRKB and TRKC (Blanchard et al., 2015). Crucially, subsets of neurons from both protocols are sensitive to capsaicin, indicative of a peptidergic C-fibre identity. However, the conversion efficiency for both protocols is low, with < 5% of original fibroblasts converting to sensory neurons. This approach does not benefit from the advantages of utilising a pluripotent intermediate such as the limitless supply of cells and ease of genome editing, but does remove the laborious, costly and variability-inducing step of cellular reprogramming. Low conversion efficiency may be a consequence of the starting cell type, as forced expression of transcription factors in SC-SN precursors shows substantially increased differentiation efficiency (Nickolls et al., 2020, Schrenk-Siemens et al., 2015). Sensory neuron specification derives from two distinct neural crest (NC) migratory waves: first, mechanoreceptors and proprioceptors are generated from NEUROG2^+^ NC cells, followed by nociceptors from NEUROG1^+^ NC cells (Marmigère and Ernfors, 2007). Viral overexpression of NEUROG2 in SC-SN progenitors substantially increases the production of MAFA^+^ low-threshold mechano-sensitive neurons (Schrenk-Siemens et al., 2015). These neurons exhibit a molecular and functional profile consistent with rapidly adapting mechanoreceptors (RA-LTMRs). Similarly, overexpression of both NEUROG2 and BRN3A in SC-SN progenitor cells generates cold-sensitive mechanoreceptors, or large-diameter RA-LTMRs, dependent on the duration of NEUROG2/BRN3A expression (Nickolls et al., 2020). Mimicking the endogenous second wave of sensory neurogenesis, overexpression of NEUROG1 in SC-SN progenitor cells promotes the differentiation of TRPV1^+^ nociceptor-like neurons (Boisvert et al., 2015).

DRG neurons are derived from the NC through embryogenesis. Despite the molecular and functional similarity of DRG and trigeminal ganglion (TG) neurons (Lopes et al., 2017a), the latter are derived from a completely distinct neuroectoderm area, the cranial placodes. A protocol for generating neurons via placodal precursors is available (Dincer et al., 2013). These TG-like neurons are likely to be of great use for studies of migraine and other headache disorders, as well as studies of somatosensation of the head and face.

## How should the identity of SC-SNs be validated?

A common challenge to all differentiation protocols is to validate how representative derived neurons are to endogenous counterparts. Sensory neuron populations are commonly classified based on their stimulus–response profiles and/or transcriptional signature. Therefore, characterising SC-SN gene expression and functional response to appropriate stimuli should be the ambition (Blanchard et al., 2015, Chambers et al., 2012, Nickolls et al., 2020, Schrenk-Siemens et al., 2015, Wainger et al., 2015). Differentiated neurons are often judged by the expression of canonical population markers as defined in mouse, yet a major rationale of SC-SN modelling is species differences. A recent study by Nickolls *et al.* provides an excellent example of why using rodent tissue as reference can be sub-optimal. Neurons were differentiated that expressed TRPM8 and PIEZO2, with corresponding functional sensitivity to both cooling and mechanical stimulation (Nickolls et al., 2020). TRPM8 and PIEZO2 expression does not overlap in mouse (Szczot et al., 2017), however, analysis of hDRG tissue revealed a substantive population of PIEZO2^+^TRPM8^+^ neurons. Single unit recordings from monkey and man (Adriaensen et al., 1983, Sumino and Dubner, 1981) and “Weber’s deception,” the phenomena that cold objects are perceived heavier than warm objects (Weber, 1851), support the hypothesis that cold mechanoreceptors are present in human but not rodent. SC-SNs from several differentiation protocols co-express P_2_X_3_R and CGRP (Chambers et al., 2012, Saito-Diaz et al., 2021, Wainger et al., 2015), despite these proteins marking largely non-overlapping populations in mouse. Recent *in situ* hybridisation studies of human DRG tissue reveal a large population of neurons expressing both markers (Shiers et al., 2020), suggesting that SC-SN are accurately replicating an endogenous sub-population and are not the result of inaccurate differentiation. Ultimately, we must be able to compare to endogenous human sensory neurons. Some SC-SN populations align well with bulk mRNA-sequencing of hDRG (McDermott et al., 2019, Young et al., 2014). The recent availability of single-cell RNA sequencing of non-human primate DRG (Kupari et al., 2021) and the soon-to-be-available human data sets (Tavares-Ferreira et al., 2021) herald an exciting time for SC-SN research, offering a “gold standard” resource for comparative transcriptomics. Also, hDRG neuron cultures could be used as a functional comparator, highlighting the potential synergy between the two cell models.

## What should SC-SNs be used for?

Much like hDRG neurons, SC-SNs provide a human translational platform to test analgesic strategies before clinical testing (Pettingill et al., 2019, Weir et al., 2017). The opportunity to create isogenic lines lacking drug targets is an especially attractive approach to validate the specificity of novel pharmacology (McDermott et al., 2019). In terms of basic biology, SC-SNs have provided valuable insight into rare genetic forms of pain. They have been used to investigate gain and loss of function mutations in Na_V_1.7 (McDermott et al., 2019, Cao et al., 2016, Meents et al., 2019), define the critical role of PIEZO2 in mechanotransduction (Schrenk-Siemens et al., 2015), and causally link mutations in the two-pore K^+^ channel, TRESK, to migraine (Pettingill et al., 2019) (for a detailed review of SC-SN use in genetic pain modelling, see (Lampert et al., 2020)). Given the rarity of these patient cohorts, SC-SN modelling of such conditions is more realistic than obtaining donor hDRG neurons. Will SC-SNs also prove valuable for the study of environmental factors of chronic pain? Unlike hDRG neurons SC-SNs are assumed to represent naïve and uninjured sensory neurons (Jones et al., 2018, Wangzhou et al., 2020), which creates an opportunity to induce injury *de novo* and compare to naïve counterparts (Baskozos et al., 2020). This approach represents a model of traumatic nerve injury and allows for nerve injury and regeneration processes to be studied. Other studies have used neurotoxins to induce nerve injury, for example, chemotherapeutic agents (Jones et al., 2018, Xiong et al., 2020). Critical to the value of these experiments is representative dosing regimen. Here, *in vitro* studies could learn from *in vivo* studies, for example, by mirroring drug concentrations measured in tissue of *in vivo* dosed animals (Li et al., 2021). Effort invested to generate representative neurons would be wasted if non-physiological treatment regimens are used. Much like there are standard protocols for inducing neuropathic pain *in vivo* (Gadgil et al., 2019), the field should aim for validated and consistent protocols of *in vitro* regimes for each agent. This would reduce both inter-lab variability and efforts of replication.

## Challenges ahead for SC-SN models of pain

Stem cell technology suffers from several technical challenges which currently require considerable experimental resource to overcome. Variability is inherent across differentiation batches, between cell lines, and when performed by multiple research groups (Volpato et al., 2018). This confounds data interpretation and makes robust experimental design imperative. Inter-line variability exists even between iPSC-derived from healthy, age and sex-matched donors. The primary cause is genetic differences (Kilpinen et al., 2017), however, inter-donor differences are greater than observed for primary DRG tissue, strongly suggesting that subtle variations in reprogramming, culture and differentiation contribute (Schwartzentruber et al., 2018b). Advances in gene editing and the opportunity to generate isogenic control lines make it possible to circumvent some of this variability. Disease mutations can be introduced into well characterised control lines, or pathogenic mutations reverted to wild-type alleles in disease lines (McDermott et al., 2019, Nickolls et al., 2020, Pettingill et al., 2019). In this regard, accessible repositories of pain-associated iPSC lines with known differentiation propensities would be a timely advance for the field. Such iPSC banks exist for a range of patient iPSC lines and these efforts demonstrate that multi-centre, multi-sector initiatives are effective ways to mitigate the volume of labour and resource required (Morrison et al., 2015). Of course, this approach is only possible in cases where pathogenic mutations are few and well-defined. To date, SC-SNs have mainly been used to model heritable painful disorders caused by highly penetrant monogenic variants (Cao et al., 2016, McDermott et al., 2019), however, these likely underlie pathology in the minority of chronic pain patients. Studying common variants of modest/low effect size, such as those proposed to contribute to more common forms of chronic pain (Calvo et al., 2019) will be a challenging task. One study has defined a disease phenotype in neurons derived from a patient with idiopathic small fibre neuropathy (SFN) (Namer et al., 2019) and a second study has demonstrated it possible to identify polygenic factors underlying inherited erythromelalgia (IEM) (Mis et al., 2019). These early examples give confidence that SC-SN modelling will be powerful enough to investigate multi-factorial pain disorders,

Endogenous human sensory neurons develop and mature over years. SC-SNs cultured for weeks/months are therefore unlikely to represent fully mature neurons. SC-SNs derived from the Chambers protocol continue to express the developmentally regulated Na_V_1.5 (Eberhardt et al., 2015) and remain small relative to hDRG neurons even after several months of culture (McDermott et al., 2019, Zhang et al., 2017). While not likely to be of concern when modelling early-onset heritable conditions such as congenital insensitivity to pain (CIP) or IEM, age is a major contributing factor to neuropathic and inflammatory pain (Bouhassira et al., 2008, Fayaz et al., 2016). How this should be considered or experimentally mitigated are open questions. SC-SNs are amenable to long-term culture (>1 year) but this is a highly costly approach. Studies to map SC-SN profiles onto the developmental trajectory of sensory neuron embryogenesis (Sharma et al., 2020) could be valuable and would allow greater transparency for the developmental state of neurons under study.

## Future perspectives: Multicellular systems

Nociception requires sensory neurons to act in concert with varied cell types and aberrant inter-cell signalling contributes to pathological pain (Finnerup et al., 2020). The opportunity to create *in vitro* systems to study cell–cell interactions is therefore attractive. Rodent sensory neurons have been co-cultured with glia, immune cells, peripheral end targets and second-order neurons (Château et al., 2007, Päiväläinen et al., 2008). The first SC-SN experiment of this kind successfully introduced rat Schwann cells, which functionally myelinate iPSC-SNs (Clark et al., 2017). Since then, iPSC-SNs have been successfully cultured with intrafusal muscle fibres to form the appropriate synaptic architecture (Mazzara et al., 2020). Cultures of hDRG neurons inherently contain other cell types present in the DRG, such as satellite glial cells (Valtcheva et al., 2016); however, no published studies have investigated interactions between hDRG neurons and exogenously applied cell types. Other co-culture systems using SC-SNs or hDRG neurons will doubtless be possible and will be a great tool to investigate cell-to-cell signalling in pain.

Homogeneous cultures likely reduce experimental variability and therefore offer greater power to observe cellular phenotypes. However, the ability, for example, to assess the effect of an analgesic candidate on multiple subtypes of sensory neurons in the same culture may be more valuable for translation. An advantage of SC-SNs is the potential to use three-dimensional organoids to create complex tissue structures that include multiple populations of sensory neuron-like cells and their interacting cellular partners. Monolayer strategies are thought to bias towards homogenous cultures, whereas cultures maintained in suspension lead to more cell type diversity and potentially better reflect endogenous neurogenesis (Chiaradia and Lancaster, 2020). Efforts to differentiate SC-SNs via three-dimensional cultures have resulted in clusters of cells termed “DRG organoids.” These structures contain 8 transcriptionally distinct sensory neuron-like subtypes, in addition to cells resembling Schwann cells and satellite glial cells (Mazzara et al., 2020). Some clusters have expression consistent with nociceptors, mechanoreceptors and proprioceptors, although the extent to which clusters represent diverse mature cells, or neurons at different stages of a developmental pathway remains to be confirmed (Mazzara et al., 2020). A recent study successfully co-induced epithelial and neural crest cells within one aggregated structure, termed a skin organoid (Lee et al., 2020). Tissue developed to include dermis, epidermis, and functional hair follicles. Organoids were richly innervated by sensory neuron-like cells and also contained S100β^+^ cells, which could represent satellite glial and/or Schwann cells. Neurite endings targeted hair follicles, reminiscent of endogenous lanceolate and circumferential endings. Finally, Merkel cells were present near neurite endings. Further work is required to profile the different cell classes and functional responses of organoids to naturalistic stimuli, but the mere presence of several different cell types involved in a mechanosensory complex is an exciting prospect.

## Conclusions

hDRG neurons and SC-SNs have distinct characteristics as experimental models, which afford advantages and disadvantages in different experimental settings (Table 2). In essence, there is no “gold standard” *in vitro* human sensory neuron model and knowledge of the strengths and limitations of each is of utmost importance. The two should not be considered competing models, but as complementary approaches that offer versatility for preclinical pain research. Differences can be leveraged, and the strength of each model enhanced by cross-validating findings between the two. There is a compelling case for an accelerated transition to human models, however, rodent sensory neurons undoubtedly remain more readily available, cheaper and require less technical knowledge. Clearing these practical hurdles will be critical to increased uptake and the full realisation of the great potential human sensory neuron models hold for advancing our knowledge of pain biology.

**Table 2:** Comparative features of hDRG and SC-SNs. Distinct features of the two models afford advantages and disadvantages in differing experimental contexts. (See below-mentioned references for further information.)

## Declaration of Competing Interest

The authors declare that they have no known competing financial interests or personal relationships that could have appeared to influence the work reported in this paper.
